# Nutrition and inborn errors of metabolism: challenges in Phenylketonuria

**DOI:** 10.1186/1824-7288-40-S1-A41

**Published:** 2014-08-11

**Authors:** Elvira Verduci, Valentina Rovelli, Francesca Moretti, Juri Zuvadelli, Elisabetta Salvatici

**Affiliations:** 1 Department of Pediatrics, San Paolo Hospital, Department of Health Sciences, University of Milan, 20142, Italy

## 

Phenylketonuria (PKU) is caused by the deficiency of the phenylalanine hydroxylase enzyme, which converts phenylalanine (Phe) to tyrosine. If left untreated from birth, this deficiency results in high levels of Phe in the blood, neurotoxic to the brain [1].The restriction of dietary Phe represents the mainstain of PKU management. PKU diet is mainly made up by variable amounts of vegetables and fruits (poor in Phe natural foods), minimal amounts of animal products (usually milk), low-protein foods (low-protein bread and pasta) and Phe-free protein substitutes, which provide mainly essential aminoacid and micronutrients, to reach the required amount of daily protein, minerals and vitamins [2,3]. This type of dietary regimen provide lower saturated and polyunsaturated fat, cholesterol as well as higher carbohydrates intake than healthy pediatric population. The PKU diet follows the norms of the so called “prudent” diet for the prevention of cardiovascular disorders. In particular saturated fats may be less than 7% and polyunsaturated higher than 5% total energy with a supply less than 50 mg cholesterol per day [2]. Indeed PKU children show lower plasma cholesterol levels as compared to healthy children, particularly low density lipoprotein particles. Nevertheless both dietary habits and genetic predisposition may interact in keeping low blood lipid levels in PKU population [2]. However a lower antioxidant status and higher homocysteine levels have been reported in PKU, suggesting a possibly increased risk for thrombosis, atherosclerosis and stroke [4,5].

Furthermore, even if PKU children are routinely long-term monitored for dietary intake some studies showed evidence for overweight in this population [6,7]. However more data on body composition in PKU individuals are needed. Further research should be necessary to better understand the nutritional quality of low-protein foods and Phe-free protein substitutes. This nutritional aspect is particularly interesting, in view of recent results about low-protein products: low-protein bread, pasta, flour and breakfast cereals appear to provide from 2 to 18% more energy than their protein-containing equivalent food [6] and some low-Phe pasta and crackers, commercially available, show an high glycemic index (data unpublished). Moreover an altered food behaviour, such as an irregular intake of higher fat food or low-protein foods, has been reported [8,9]. Few data are available on physical activity levels in PKU patients [8].

In conclusion given the growing population of adults with PKU, it could be important to investigate the non-communicable diseases risk in this population to better optimize nutritional treatment strategies.
